# Achieving sustainable, environmentally viable, solarized vaccine cold chain system and vaccination program—an effort to move towards clean and green energy-driven primary healthcare in Lebanon

**DOI:** 10.3389/frhs.2024.1386432

**Published:** 2024-06-06

**Authors:** Bhrigu Kapuria, Randa Sami Hamadeh, Farah Mazloum, Joe Akl Korbane, Kyaw Aung, Doaa Kamal, Nariman Chamoun, Sabin Syed

**Affiliations:** ^1^ UNICEF Lebanon Country Office, Beirut, Lebanon; ^2^ Ministry of Public Health (MoPH), Beirut, Lebanon

**Keywords:** solarization, vaccine cold chain system, sustainable, vaccine management, environment friendly

## Abstract

**Introduction:**

Lebanon faces severe economic and energy crises, impacting its healthcare system, particularly vaccine storage. Traditional gas or kerosene-powered refrigerators often fail to maintain necessary temperatures for vaccine efficacy. This study explores transitioning to solar direct-drive (SDD) refrigerators to ensure reliable vaccine storage.

**Methods:**

A multi-phase methodology was employed, beginning with an inventory assessment of existing cold chain equipment. The implementation involved stepwise replacement of identified refrigerators across health facilities, including Primary Health Care Centers (PHCCs) and dispensaries. Feasibility, cost-effectiveness, and environmental impact were evaluated.

**Results:**

Findings indicate that solarization significantly reduces vaccine wastage, ensures stable temperatures, and cuts operational costs by decreasing dependence on non-renewable energy sources. Over 1,000 SDD units were installed across more than 800 health facilities. Additionally, PHCC solarization improved vaccine preservation and enhanced the resilience of health services overall.

**Discussion:**

The solarization initiative demonstrates the critical role of renewable energy in strengthening healthcare infrastructure, especially in crisis-hit regions. Solar-powered systems provide a reliable and sustainable solution for vaccine storage, reduce carbon footprints, and build public trust in the immunization system. Challenges included geographical and structural limitations, which were addressed through comprehensive planning and collaboration with local stakeholders. Solarization of Lebanon's vaccine cold chain and PHCCs marks a significant step towards sustainable and resilient healthcare infrastructure. The model offers a robust framework for other regions facing similar economic and energy challenges, highlighting the importance of renewable energy solutions in healthcare.

## Introduction

Lebanon is currently battling one of its worst economic crises in decades. The country defaulted on its national debt in 2020, and its currency has collapsed in value. An acute energy crunch is a compounding problem, with households nationwide grappling with long power cuts and increased fuel prices. The state-run utility Electricite du Liban (EDL), which accounts for about 90% of the country's electricity production, has been plagued by dire cash shortages, and has only been able to provide power for a few hours a day normally ranging from 2 to 4 h of grid electricity per day even in urban cities. Faced with power outages, the Lebanese healthcare system, including the vaccine supply chain management, has been left in a vulnerable situation with an immediate need for alternatives. Lebanon provides public immunization services through 800+ health facilities comprising of 275+ Primary Health Care Centers (PHCC) and 500+ Dispensaries. Solar direct-drive (SDD) refrigerators and freezers can serve as a good option for vaccine storage in areas without reliable electricity, and many models are now WHO-prequalified, feeding into the reliability of these equipment. Additionally, the nation's economic turmoil coupled with the surge in fuel prices, severely restricts the practicality of utilizing costly private diesel generators as a backup power source for electricity. Considering the current scenario, moving towards a sustainable solar-driven energy solution can bail out the system from crisis as well as strengthen it for upcoming years to face any possible parallel challenge. Also, the economic and fuel crisis potentially impact the immunization and other essential primary healthcare services as well, so along with solarization of the cold chain equipment another important step to safeguard vaccination programs was to solarize the PHCCs after careful feasibility assessment.

Furthermore, an Effective Vaccine Management Assessment (EVMA) was conducted through the first quarter of 2021 by a team of trained EVM country managers and assessors, the assessment has brought to light strengths and weaknesses of the Immunization Supply Chain (iSC) system in Lebanon. While EVM recommends a minimum score of 80% for all E-Criteria, the overall national EVM score for Lebanon for 2021 was 63%, with E5 (Maintenance and Repair) and E2 (Temperature Management) score being the lowest, this highlights the importance of assessing the cold chain equipment replacement needs, specifying preferred models and type of power source, to enhance and establish dependable cold chain systems that can effectively support vaccination programs using standardized cold chain equipment according to WHO PQS standard. The cold chain inventory assessment indicated that almost all cold chain equipment used at health facilities were not designed for vaccine storage and were not WHO Prequalified for vaccine management.

Covid-19 Pandemic emphasized the vulnerability of vaccine cold chain systems as well as their subsequent ill effect on overall vaccination programs, interrupted power supply poses a greater challenge on efficient functioning of cold chains, hence the potency of vaccines and furthermore on the desired effect of immunization programs, it was deemed necessary to work towards solutions that are self-reliant and sustainable in case of any other parallel emergency in future.

### Literature review

Solar energy provides a sustainable and renewable source of power. This means that once the initial installation costs are covered, running costs can be minimal, particularly in regions with consistent sunlight (1). Solar systems require maintenance to ensure optimum performance. In remote or resource-limited settings, maintenance can be challenging. Training local staff and creating a network of skilled technicians can help mitigate this challenge (2). In areas with prolonged periods of low sunlight, there is a need for efficient battery storage systems to ensure a continuous power supply. Advancements in battery technology, like lithium-ion and flow batteries, have shown promise in these situations (3). A project in Chad aimed to introduce solar-powered refrigerators for vaccine storage. The project reported reduced vaccine wastage and increased vaccination coverage in the targeted regions (Martin et al., 2017) (4). India embarked on a mission to solarize its primary health centers. This initiative not only improved vaccine storage but also enhanced the overall quality of healthcare services in rural areas (Rao & Sinha, 2019) (5).

### Objective

The paper aims to summarize how solar energy was used to safeguard vaccine storage and support the vaccination process, along with discussing the course of assessing the feasibility and highlighting the benefits of implementing a solarized vaccine cold chain system in Lebanon.

## Methodology

**Figure 1:**
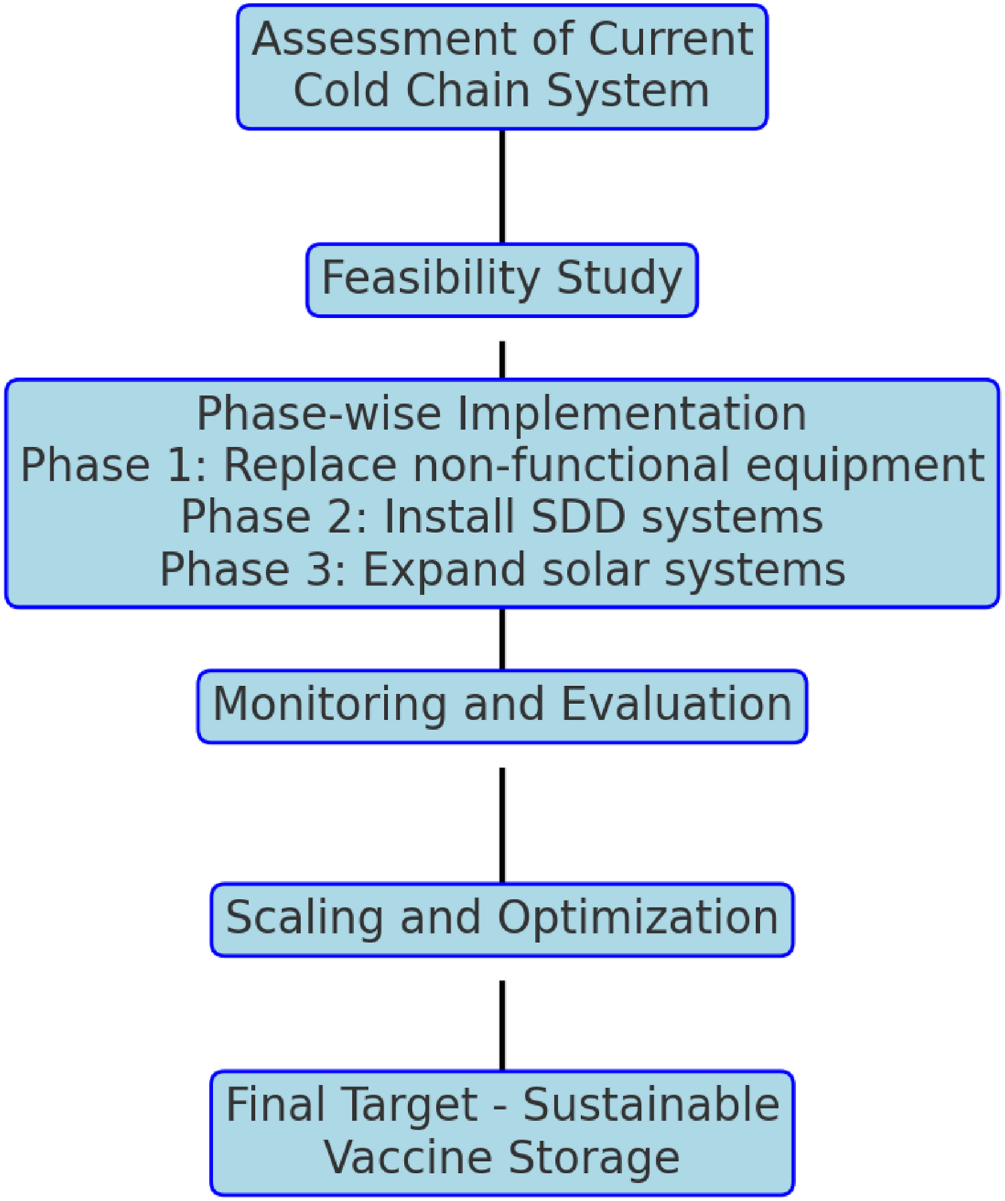
Process flow for achieving sustainable vaccine cold chain system.

MoPH and UNICEF followed a two-level approach i.e., solarization of vaccine cold chain system through cold chain inventory assessment considering the considerations and recommendations of the EVM assessment 2021 (Figure 1). The intent was to safeguard not only the vaccine supply chain ensuring the availability of vaccines but also to protect immunization and essential public health programs.

Lebanon climates support solar irradiance for solar vaccine refrigeration aptly, making the choice of solar technology and its implementation an advisable solution for a sustainable, reliable, and cleaner cold chain system. With the near total absence of grid electricity in the country, rising fuel cost and the dependency on diesel operated generators, solar cold chain equipment was considered the right choice. A facility-level cold chain inventory analysis was conducted to identify the cold chain equipment status and identify gaps and further. Based on the findings of cold chain inventory assessment, the implementation of replacing the identified refrigerators, comprised of three prioritization phases approach, whereby, the first phase was to replace nonfunctional or sick equipment given priority by upgradation followed by replacement of domestic fridges or non-standardized cold chain equipment being operated on generators or gas while complete updating the entire cold chain network working of solar energy, wherever feasible, by June 2023.

In addition to the cold chain inventory assessment, analysis of the inventory gap assessment of the EVM report 2021was considered to conduct the solarization of cold chain equipment at Lebanon, the assessment report brought focus on the requirement of procuring additional and/or replacement CC equipment for Dispensaries, replacement non-WHO PQS equipment with WHO PQS equipment, replacement of obsolete (old) equipment (e.g., equipment older than 10–12 years)A facility-level cold chain inventory analysis was conducted to identify the cold chain equipment status and gaps, along with additional requirements. Based on the findings of this inventory assessment, a prioritization plan was created, with non-functional or ailing equipment designated for immediate upgrade, followed by the replacement of domestic fridges and non-standardized electric or gas-operated cold chain equipment. The overarching objective was to comprehensively update the entire cold chain network by June 2023, to operate on solar energy wherever feasible.

In addition to the cold chain inventory assessment, an analysis of the inventory gap assessment from the EVM report in 2021 was considered in the context of the solarization of cold chain equipment in Lebanon. The assessment report highlighted the need to procure additional and/or replacement Cold Chain equipment for dispensaries, replace non-WHO PQS equipment with WHO PQS-compliant equipment, replace obsolete equipment (more than 10–12 years old), address non-functioning equipment that could not be economically repaired, and acquire additional equipment to address storage volume shortages. As part of the comprehensive cold chain network update, cold rooms at central warehouses and district vaccine stores were solarized wherever feasible, and two regional cold rooms were established to operate exclusively on solar energy.

As a second step towards a more sustainable public primary health care services at PHCCs was solarization. As such, UNICEF conducted a “Feasibility and Energy Need Assessment” for all 275 PHCCs to identify PHCCs fit for solarization. The activity was conducted between December 2022 and January 2023. The assessment covered all components related to solarization, including space for solar panels, rights to access and use rooftop by the health facility (if in rented/shared building), electrification and equipment. A detailed facility assessment template was developed, and an agency engaged in solarization was selected to visit every PHCC and complete the assessment. The evaluation team was comprised of members with skills and knowledge related to solarization and electrical engineering. The findings from assessment results showed that 15 PHCCs cannot be solarized because of either limited space or no permission to use roof for Solar PV Panels installation and 159 were found fit for solarization. In Lebanon's context, emphasis was given on procuring from the local markets and establishing Long Term Agreements (LTA) with solarization companies. UNICEF was able to establish LTAs with four solar companies with a three-year validity, in the PHCCs solarization process.

### Key success areas of moving towards solarization of vaccine cold chain and PHCCs

UNICEF Lebanon with the support from MoPH and developmental partners has been involved solarization of the vaccine cold chain system through procurement and installation of over 1000 SDDs across 800+ health facilities, solarizing 14 district vaccine stores, central and regional cold rooms along with solarization of 150 PHCCs providing immunization and primary health care services; thus, creating a sustainable, reliant, environment friendly and trustworthy immunization system in the country. The solarization of 150 Primary Health Care Centers (PHCCs) contributed to the enhancement of the entire PHCC network across the country by transitioning to solar energy wherever it was feasible.

The effort has led to the establishment of a network of solar-powered cold chain, extending from the national level down to the final distribution point, wherever it is practically achievable. Along with the solarization process, installation of Remote Temperature Monitoring Devices was carried out for further real time monitoring of the vaccine supply chain and to more effectively address any issues.

### Reduced cost of ownership

The SDD refrigerator would have the lowest total cost of ownership in areas with unreliable electricity and suitable solar irradiance, and where a solar service provider can be funded to provide the necessary support.

Substantive energy cost savings are anticipated through the solarization of Primary Health Care system in the country. It has not only saved thousands of dollars for health centers in vulnerable locations allowing them to serve families in their areas, but also reduced the country's carbon footprint by reducing the need for diesel-operated generators.

### Sustainable vaccine cold chain system

Solarization of vaccine cold chain systems eliminates the need to depend on diesel generators or use of outdated and non-standardized fridges operating on gas or kerosene for vaccine storage. Installation of SDDs and solarization of PHCCs eventually brings about a paradigm shift in the chief source of energy in smooth functioning of the vaccine cold chain system across all levels.

### Minimizing the risk of exposing the vaccines to freezing temperatures

Keeping the temperature within the acceptable range of +2°C to +8°C for vaccines is difficult in absorption refrigerators (refrigerators powered by gas or kerosene), there is a high risk of exposing vaccine to freezing temperatures. Therefore, in a country like Lebanon which is undergoing electricity crisis, utilization of solar energy becomes a more viable, dependent, and superior option.

### Cleaner and greener source of energy

The solar powered systems will have a direct and positive impact on the environment and climate as well as lead to significant savings, in the form of diesel expenses for the generators, as the essential loads of PHCCs will run on solar energy.

Solarization of the immunization cold chain system is a pioneering initiative that merges healthcare and sustainable energy solutions. By harnessing the renewable resources, this approach not only ensures a continuous and reliable source of energy for vaccine storage but also diminishes the dependency on conventional fuels. This transition significantly reduces carbon emissions, contributing to a cleaner and healthier environment. Furthermore, leveraging solar energy showcases a commitment to sustainable practices, setting a benchmark for other sectors to follow. Overall, it exemplifies a holistic strategy towards clean energy utilization in critical healthcare infrastructure.

### Building public trust in public immunization system

Traditionally Lebanon's immunization system has remained more dependent on the private health care providers, however due to the ongoing financial instabilities, the immunization services have become more and more inaccessible for the general population, leaving them more vulnerable to multiple VPDs. MoPH along with other development partners have been continually working towards bringing about a change in the immunization seeking behavior of the people through public sector. Solarization of cold chain systems for vaccines and PHCCs, has been a major contributing factor in building the trust of people in the government immunization delivery system, through ensuring the safe storage of vaccines at recommended temperature ranges, and uninterrupted availability of services.

### Challenges of solarization of cold chain system

The chief limitation faced during solarizing any cold chain system at any level and PHCCs is that there were areas which either due to geographical reasons or the constructional reasons, have cold chains situated on floors or regions which might not experience the desired amount of sunlight for smooth functioning of the solar cold chain systems.

Another challenge encountered during the installation of solarized cold chain in Lebanon, was the fact that some health facilities were located in rented locations where owner has reservations to allowing solar panel installation or health facility located in multistory building leading to limited space to install solar panels.

Accessibility in some of the buildings for installing, maintaining, and securing the equipment was an additional challenge faced by the system.

### Outcome

UNICEF Lebanon with support from MopH and developmental partners has been involved in solarization of the vaccine cold chain system through procurement and installation of over 1,000 SDDs across 800+ health facilities, solarizing 14 district vaccine stores, central and regional cold rooms along with solarization of 150 PHCCs providing immunization and primary health care services; thus, creating a sustainable, reliant, environment friendly and trustworthy immunization system in the country. The solarization of 150 PHCCs supported in upgrading the entire PHCC network in country on solar energy, wherever feasible.

The initiative has resulted in forming a continued chain of solarized cold chain from the National level till the last distribution point wherever feasible. Along with the solarization process, installation of Remote Temperature Monitoring Devices was carried out for further real time monitoring of the vaccine supply chain.

## Discussion

The World Health Organization estimates that up to 50% of vaccines are wasted globally every year (6); a large part is because of lack of temperature control and the logistics to support an unbroken cold-chain. The need is to develop resilient, reliable, and sustainable cold chains to prevent valuable loss, and solarization of vaccine cold chain equipment cannot be emphasized enough in this regard (7).

Lebanon embarked on the journey to solarize nearly the entire cold chain network, wherever feasible, from national stores to last fridge storing vaccines due to prevailing circumstance in the country, that eventually led to creation of a reliable vaccine storage chain with viability for nearly a decade, shielding vaccines from ongoing disruptions in the country due to economic and electricity crisis (8). In doing so, Lebanon also became one of the few countries in the world managing nearly the entire vaccine storage system on clean and green solar energy.

Comparable to Lebanon, there are efforts across the globe like the Rwanda Cooling Initiative (RCOOL) by the Rwandan Government and the United Nations Environment Program's United for Efficiency (U4E) (9) team provides the foundation for the new Centre. Through RCOOL, Rwanda's Cabinet released a National Cooling Strategy in 2019 calling for concrete action on sustainable cold chains.

Planning activities must consider the time, tools, and resources that technicians require to properly install an SDD refrigerator. Minimizing the need for last-minute installation-related procurements and planning for those that cannot be anticipated, will be critical for ensuring smooth deployments in the future of smooth installation of SDDs (10).

Based on current operating hours of PHCCs and fuel cost to run generators, the 150 PHCC-solarization will save approximately USD 700,000 every year, each facility saving on average USD 4,700 per year. This means that in approximately 2.5 years a full return for the initial investment of USD 1,900,000 will be achieved.

For the general population, the solarization initiative will lead to a cost saving of USD 1.56 per person utilizing PHCCs, which represents a 9% reduction in the average out-of-pocket primary health expenditure per capita. The average out-of-pocket primary health expenditure in Lebanon was approximately USD 17 in 2022, and around 855,000 people frequented PHCCs.

The role played by supportive infrastructure for the cold chain and PHCCs such as a regular supply of electricity cannot be underplayed. In this regard, there is a need to improve electrification, especially in the last mile, for which the potential of solar-driven technology must be explored to integrate sustainable development (11). The functioning of health centers can be solarized to tackle the issue of regular power outages, this may significantly reduce the disruption in service provision and increase the uptake of services.

### Way forward

The role of solarization in creating self-reliant vaccination systems can be well appreciated into the entire health care domain, especially in struggling economies like Lebanon. There is an appetite for power beyond the cold chain, medical officers and users expressed their desire for electricity availability for all health facilities including PHCCs and Dispensaries. Emerging “energy harvesting” technologies being developed by manufacturers and global partners could serve this need well, by utilizing excess energy from the SDD for other health purposes, such as lighting, cell-phone charging, etc.

There is broad consensus that electrifying health-care facilities with clean and sustainable energy is fundamental for a healthier world (12). It is also increasingly recognized as key to advancing economic, environmental and climate outcomes. There is thus an urgent need to improve the geographic coverage, quality and frequency of data collection on energy access in health-care facilities. With an integrated tracking system that routinely collects comprehensive and standardized electricity access indicators, countries will be able to monitor progress towards powering health-care facilities in a robust and cost-effective way (13). This, in turn, will allow assessment of the impacts of electricity access on health, climate and other development goals; forecasting of future needs; and better allocation of limited resources. Such a system would enable better knowledge sharing between the health, energy and other sectors. Common challenges across settings and opportunities to collaborate can be identified. Further dissemination of experiences with new and innovative energy solutions and other lessons learned to policy-makers and practitioners would maximize evidence-informed policies.

The solarization of the cold chain system and Primary Health Care centers marks the initial step in advancing solar technology across essential domains, encompassing basic amenities such as lighting, ventilation, information and communication technologies, and life-saving medical devices. This extends beyond safeguarding the cold chain for the secure preservation of vaccines, blood, and critical medicines that necessitate refrigeration. Significantly, proper storage practices also help prevent vaccine wastage (14).

The utilization of solar energy plays a pivotal role in climate change mitigation efforts. Shifting public health systems towards solar power substantially reduces the carbon footprint of healthcare services (15). The lessons learned from Lebanon serve as a compelling example, highlighting the imperative to solarize primary healthcare centers. This ensures the uninterrupted delivery of fundamental services in the face of crises, whether natural or human-induced. A robust public health system is essential for effectively responding to future pandemics and epidemics, and investing in solar energy represents a crucial step in fortifying our primary healthcare network (16).

### Solarization of dispensaries

Considering the benefits and based on availability of resources, Lebanon will further move towards solarization of dispensaries. A network of 500 + dispensaries providing immunization services along with other health services are to be solarized as per feasibility.

UNICEF conducted a “Feasibility and Energy Needs Assessment” for all Dispensaries to identify potential health facilities needing support. This will further ensure uninterrupted health care services across various geographies within the country. Due to the current economic and energy crises, dispensaries are not able to maintain the full functionality of, the nearly total absence of grid electricity and high fuel prices (to supply diesel generators) are major contributing factors, UNICEF is supporting the solarization of these dispensaries and aiming towards an environmentally friendly, financially viable and sustainable primary healthcare system.

## Conclusion

In conclusion, the solarization of the vaccine cold chain system and PHCCs in Lebanon is a move towards clean and green energy that can help to address the current economic crisis and energy crunch in the country. The use of solar direct-drive refrigerators and freezers can provide a sustainable and reliable solution for vaccine storage in areas without reliable electricity (17). The implementation of this system requires sufficient solar energy, the presence of a solar service provider, and secure funding. The key success areas of moving towards solarization of the vaccine cold chain system include reduced cost of ownership, sustainable vaccine cold chain system, minimizing the risk of exposing vaccines to freezing temperatures, cleaner and greener source of energy, and building public trust in public immunization systems (18). Further research is needed to explore the feasibility and effectiveness of solarization of the vaccine cold chain system and primary health care centers in Lebanon and other countries facing similar challenges.

## Data Availability

The original contributions presented in the study are included in the article/Supplementary Material, further inquiries can be directed to the corresponding author.
